# Characteristics of Akinetic and Dyskinetic Left Ventricular Aneurysms in the Context of Echocardiographic Diagnosis and Treatment Selection

**DOI:** 10.3390/medicina60071141

**Published:** 2024-07-16

**Authors:** Slobodan Tomić, Stefan Veljković, Dragana Radoičić, Olivera Đokić, Armin Šljivo, Ivan Stojanović, Aleksandra Nikolić, Milovan Bojić

**Affiliations:** 1 Cardiovascular Institute “Dedinje”, 11040 Belgrade, Serbia; bobantomic99@gmail.com (S.T.); dragana.kastratovic@yahoo.com (D.R.); oljaisara@gmail.com (O.Đ.); ivan0stojanovic@gmail.com (I.S.); nikolicdrsasa@gmail.com (A.N.); dedinje@ikvbd.com (M.B.); 2 Faculty of Medicine, University of Banja Luka, 78000 Banja Luka, Bosnia and Herzegovina; 3 Clinical Center of University of Sarajevo, 71000 Sarajevo, Bosnia and Herzegovina; sljivo95@windowslive.com; 4 Faculty of Medicine, University of Belgrade, 11040 Belgrade, Serbia

**Keywords:** aneurysm, cardiac volume, cardiac surgery, echocardiography, Serbia

## Abstract

*Background and Objectives*. Distinct pressure curve differences exist between akinetic (A-LVA) and dyskinetic (D-LVA) aneurysms. In D-LVA, left ventricular (LV) ejection pressure decreases relative to the aneurysm size, whereas A-LVA does not impact pressure curves, indicating that the decrease in stroke volume (SV) and cardiac output is proportional to the size of dyskinesia. This study aimed to assess the frequency of A-LVA and D-LVA, determine aneurysm size parameters (volume and surface area), and evaluate predictive parameters using echocardiography in A-LVA and D-LVA. Furthermore, it aimed to compare individual echocardiographic parameters, according to ejection fraction (EF) and SV, with hemodynamic events shown in experimental models of A-LVA and D-LVA and their significance in everyday clinical practice. *Materials and Methods*. This clinical study included patients with post-infarction left ventricular aneurysm (LVA) admitted to the cardiovascular institute ‘’Dedinje”, Serbia. Echocardiographic volume and surface area of LV and LVA were determined (by the area–length method) along with EF (by Simpson’s method). *Results*. A-LVA was present in 62.9% of patients, while D-LVA was present in 37.1%. Patients with D-LVA had significantly higher systolic aneurysm volume (LVAVs) (94.07 ± 74.66 vs. 51.54 ± 53.09, *p* = 0.009), systolic aneurysm surface area (LVAAs) (23.22 ± 11.73 vs. 16.41 ± 8.58, *p* = 0.018), and end-systolic left ventricular surface areas (LVESA) (50.79 ± 13.33 vs. 42.76 ± 14.11, *p* = 0.045) compared to patients with A-LVA. The ratio of LVA volume to LV volume was higher in the D-LVA in systole (LVAVs/LVESV). The end-diastolic volume of LV (LVEDV) and end-systolic volume of LV (LVESV) did not significantly differ between D-LVA and A-LVA. EF (21.25 ± 11.92 vs. 28.18 ± 11.91, *p* = 0.044) was significantly lower among patients with D-LVA. *Conclusions*. Differentiating between A-LVA and D-LVA using echocardiography is crucial since D-LVA causes greater hemodynamic disturbances in LV function, and thus surgical resection of the aneurysm or LV reconstruction must have a positive effect regardless of myocardial revascularization surgery.

## 1. Introduction

Left ventricular aneurysm (LVA) is a complication of acute myocardial infarction characterized by localized, well-defined ventricular wall dilation and, according to recent reports, develops in less than 5% of patients [1]. In its acute phase, the dilation will demonstrate paradoxical movements of the ventricular wall during systolic expansion (dyskinesia) as opposed to the contraction of normal surrounding tissue. In chronic aneurysms, the aneurysmal tissue gradually transforms into dense fibrotic tissue and becomes extremely firm and relatively non-expansive (akinetic). The degree of left ventricular enlargement, according to reports, primarily depends on the size of the LVA formed over time [2]. Results of paradoxical movement of non-contractile myocardium (dyskinesia) lead to additional work for the remaining cardiac muscle, thus reducing ventricular stroke volume (SV), flow, and cardiac output, which can lead to heart failure, arrhythmias, and the risk of embolism and mural thrombus [3,4]. The non-aneurysmal portion of the left ventricle undergoes remodeling, increasing in volume and wall thickness due to secondary hemodynamic stress imposed by akinetic or dyskinetic aneurysms. The larger the ventricular volume, the greater the increase in systolic wall tension, which is one of the main determinants of myocardial oxygen consumption [5].

Current literature indicates that chronic fibrotic aneurysms primarily affect ventricular performance by causing the loss of contractile tissue. In such cases, the degree of ventricular extension, or “lost work” compared to a normal left ventricle is minor, leading to its characterization as an anatomic aneurysm. In contrast, aneurysms composed largely of scar tissue and viable myocardium or thinned scar tissue exhibit mechanical defects, including paradoxical expansion and reduced effective contractility, and are referred to as functional aneurysms [6]. Quantifying the degree of mechanical deficiency caused by dyskinetic and akinetic aneurysms is crucial for comprehending acute and chronic hemodynamic changes post-myocardial infarction and guiding appropriate patient selection for medical therapy or surgical interventions like aneurysmectomy.

**Figure 1:**
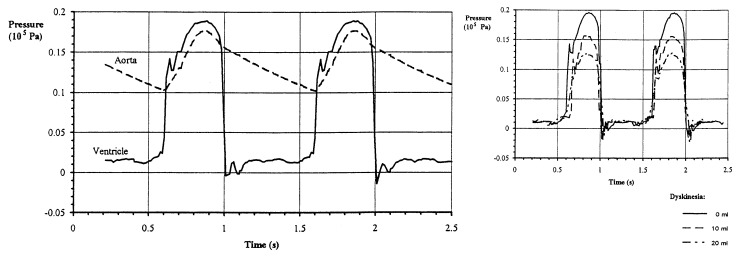
Pressure–time curves in the model of the healthy heart ventricle and aorta at 60 beats per minute and stroke volume of 70 mL. (**left**) and ejection pressure–time curves in the model of the ventricle with varying sizes of dyskinetic aneurysm. (**right**) (Source: Holger Hadlan [7]).

Holger Hadland and colleagues [7] demonstrated that the size or volume of the aneurysm does not affect the ventricular and aortic ejection pressure curve when the aneurysm is akinetic and does not cause significant mechanical deficiencies in chamber function. However, the impact of dyskinetic aneurysms on ventricular function reveals that intraventricular pressure curves created in a healthy heart and with dyskinetic aneurysms result in a proportional reduction in ejection pressure corresponding to the size of the dyskinetic aneurysm (Figure 1). Moreover, it has been shown that the loss of cardiac output is directly associated with the extent of dyskinesia rather than the surface area of the aneurysm; in other words, a large akinetic aneurysm does not impede output, but a small aneurysm with a large dyskinetic volume will increase mechanical defects and reduce output independently of the myocardial surface area. These data have demonstrated that ventricular pressure is associated with dyskinetic volume, with minimal influence from heart rate.

Holger Hadlan [7] further demonstrates that the main decrease in ventricular function primarily occurs when the aneurysm undergoes paradoxical systolic expansion, directly correlated with the compliance of the aneurysmal segment. The loss of stroke volume would be directly proportional to the amount of fluid “lost” by the expansion of the aneurysm. Paradoxical expansion of the aneurysm compared to an akinetic aneurysm is considered a predictor of surgical success [8,9,10]. Paradoxical expansion of the aneurysm, according to the hypothesis, leads to the ejection of a portion of the stroke volume into the aneurysm, thus creating volumetric stress. Several prospective studies have addressed the clinical importance of this [9]. It has been noted that not all patients with left ventricular aneurysm benefit from surgery [11,12]. Studies show improvement in functional class, exercise tolerance, and cardiac characteristics (performance) in patients with dyskinetic groups compared to those with akinetic groups. Among several preoperative and procedural variables representing risk factors for early mortality, excision of akinetic (18%) rather than dyskinetic aneurysms (8%) was suggested [13]. This suggests that paradoxical systolic expansion may prove valuable in selecting suitable patients for aneurysm resection [9,10]. Patients with dyskinetic LVA have more compromised left ventricular function preoperatively than those in the akinetic group. Patients with the best preoperative left ventricular characteristics (performance) have less benefit from surgery (akinetic group) [9,12].

This study aimed to assess the frequency of akinetic left ventricular aneurysms (A-LVA) and dyskinetic left ventricular aneurysms (D-LVA) using echocardiography, determine the parameters of aneurysm size, aneurysm volume (LVAV), and aneurysm surface area (LVAA) in systole and diastole for both types of aneurysms, determine left ventricular function parameters, end-diastolic volume (LVEDV) and end-systolic volume (LVESV), and chamber surface area in end-diastole (LVEDA) and end-systole (LVESA), investigate the relationship between aneurysm volume—surface area and left ventricular volume—surface area and their significance in assessing aneurysm size and compare individual echocardiographic parameters (ejection fraction—EF and SV) with hemodynamic events demonstrated in experimental models of akinetic and dyskinetic aneurysms and assess their significance in everyday clinical practice. The study also aimed to establish a foundation and guidelines for future research that would involve a larger number of participants to validate and expand upon the findings of this study.

## 2. Materials and Methods

This observational study included patients with post-infarction LVA admitted to the cardiovascular institute ‘’Dedinje’’, Belgrade, Serbia, for a routine transthoracic echocardiographic exam. The inclusion criteria were (i) patients diagnosed with post-infarction LVA who were admitted for routine transthoracic echocardiographic examination, (ii) patients aged 18 years or older, and (iii) those who had the ability to provide informed consent to participate in the study. Ethical approval for the study was obtained from the Bioethical Committee of Institut “Dedinje” to ensure compliance with ethical guidelines and proper research procedures. The investigation adhered to the relevant provisions of the Helsinki Declaration, which outlines ethical principles for medical research involving human participants, with a focus on safeguarding patient rights, privacy, and confidentiality throughout the study period.

### 2.1. Data Collection

Patients underwent a transthoracic echocardiographic examination in the left lateral decubitus position according to the standard protocol. Left ventricular aneurysm volume (LVAV) was measured in systole (LVAVs) and diastole (LVAVd) from apical views using the area–length method (mL). The aneurysm surface area (LVAA) was measured in systole (LVAAs) and diastole (LVAAd) from apical views and calculated planimetrically (cm^2^). End-diastolic and end-systolic left ventricular volumes (LVEDV and LVESV) were calculated using the area–length method (mL). End-diastolic and end-systolic left ventricular surface areas (LVEDA and LVESA) were calculated planimetrically (cm^2^). Left ventricular ejection fraction (EF) was determined using Simpson’s method (%). A left ventricular aneurysm was classified as “dyskinetic” if the absolute motion of adjacent chords was less than zero and as “akinetic” if the motion was equal to zero.

### 2.2. Statistical Analysis

Data were analyzed using the standard statistical software SPSS version 22, and a significance level of 0.05 was used for all applied analytical methods. Numeric features were described using classical descriptive statistical methods, including mean values and standard deviation as measures of variability. The distribution of numerical variables was assessed using the Kolmogorov-Smirnov test to determine normal distribution, and for variables meeting this criterion, parametric methods were applied. Baseline characteristics and mean values of observed features were compared using the Chi-square test, Student’s t-test, and Mann–Whitney test as appropriate.

## 3. Results

Out of 54 patients, of whom 36 (66.7%) were males with a mean age of 63.19 years and 18 (33.3%) were females with a mean age of 65.06 years, A-LVA was present in 34 patients (62.9%), while D-LVA was present in 20 patients (37.1%).

Patients with D-LVA had significantly higher systolic aneurysm volume (LVAVs) (94.07 ± 74.66 vs. 51.54 ± 53.09, *p* = 0.009), systolic aneurysm surface area (LVAAs) (23.22 ± 11.73 vs. 16.41 ± 8.58, *p* = 0.018), and end-systolic left ventricular surface areas (LVESA) (50.79 ± 13.33 vs. 42.76 ± 14.11, *p* = 0.045) compared to patients with A-LVA, although the other measured parameters of volume and surface area were also higher in this group. All other characteristics according to volumes and surface areas of the aneurysm and left ventricle in systole and diastole in the presence of A-LVA and D-LVA are presented in Table 1.

There is a higher ratio of aneurysm volume to left ventricular volume in systole (LVAVs/LVESV) in the dyskinetic group due to significantly larger values of aneurysm volume in systole (LVAVs). The relationship between aneurysm volume and left ventricular volume and the ratio of aneurysm surface area to left ventricular surface area in systole and diastole is presented for A-LVA and D-LVA in Table 2.

Patients with D-LVA had significantly lower EF (21.25 ± 11.92 vs. 28.18 ± 11.91, *p* = 0.044) compared to those with A-LVA. There was no significant difference in end-diastolic and end-systolic left ventricular volumes between the compared groups, although the end-diastolic volume showed higher values in D-LVA. The values of EF, end-diastolic, and end-systolic left ventricular volume in A-LVA and D-LVA are presented in Table 3.

## 4. Discussion

The incidence rates of A-LVA and D-LVA vary according to the literature. Couper [13] indicates in their study that out of 303 patients (over a 17-year period) who underwent aneurysm resection, D-LVA was found in 180 patients and A-LVA in 121 patients. Similarly, in another study with 87 analyzed patients, 37 (43%) had D-LVA and 50 (57%) had A-LVA [14], mirroring our findings.

Aneurysm size parameters (surface area and volume) and left ventricular function parameters (surface area and volume), besides quantifying aneurysm size, also determine the choice of surgical technique in cardiac reconstruction. In Danchin et al.’s study [15], among 18 patients divided into two groups based on clinical status, the echocardiographically measured average aneurysm surface area was 37.4 cm^2^ for the group with NYHA III-IV symptoms (dyspnea, fatigue, fainting, weakness, pretibial edema, palpitations, in instances with less than normal physical activity in NYHA III and with minimal physical activity or even rest and severe limitation of functional status in NYHA IV) and 21.2 cm^2^ for the group with good clinical findings. In Marchenko et al.’s study [16] involving 158 patients with post-infarction aneurysms, surgical treatment was determined based on preoperative echocardiographic measurements of the left ventricle and aneurysm surface areas, and the surgeon measured the intraoperative scar area of the left ventricle or aneurysm. Surgical techniques included linear plastic (Cooley), the septal technique by Stone for anteroseptal aneurysms, and endoventriculoplasty with a synthetic “patch” according to the Dor procedure. The average left ventricle surface area and aneurysm surface area varied across these techniques as follows: linear plastic (164 cm^2^ and 32 cm^2^), septal technique (186 cm^2^ and 42 cm^2^), and endoventricular plastic (217 cm^2^ and 49 cm^2^). Intraoperative scar area measurements averaged 29 cm^2^ for linear plastic, 45 cm^2^ for septal plastic, and 64 cm^2^ for endoventriculoplasty. Preoperative LVEDV ranged from 189 mL for linear plastic and 199 mL for patients with septal plastic to 251 mL for endoventriculoplasty [16].

In true left ventricular aneurysms, Di Donato [17] provides echocardiographically determined mean values of LVEDV of 216 mL and LVESV of 150 mL. The preoperative echocardiographic assessment of LVEDV with a present aneurysm averaged 261 mL and LVESV 133 mL in a study involving 41 patients [18]. Marsan and colleagues [19], in a study involving 60 patients, obtained average values of LVEDV of 247 mL and LVESV of 180 mL, according to the Simpson method. In our study, the average LVEDV in A-LVA was 261.71 mL and 286.40 mL in D-LVA. The values of LVEDV are approximate or slightly higher compared to several comparable studies [16,18,19]. The average LVESV was 207.68 mL in A-LVA and 228.95 mL in D-LVA. These values are slightly higher compared to those in a study by Marsan and colleagues [20], but significantly higher compared to those in other studies [16,18]. The LVEDA was 53.50 cm^2^ in A-LVA and 57.78 cm^2^ in D-LVA, while the LVESA averaged 42.76 cm^2^ in A-LVA and 50.79 cm^2^ in D-LVA. These values are slightly higher compared to those in a comparable study by Sobkowitz [21].

The average volumes of aneurysms in diastole were 68.79 mL in A-LVA and 83.05 mL in D-LVA, which are higher compared to the findings of Sobkowitz and colleagues (average diastolic volume 50 mL [21]. The volume of the aneurysm in systole was 51.54 mL in A-LVA and 94.07 mL in D-LVA, which is a statistically significant difference. The surface area of the aneurysm in diastole was 19.74 cm^2^ (A-LVA) and 21.67 cm^2^ (D-LVA), while in systole, it was 16.41 cm^2^ (A-LVA) and 23.22 cm^2^ (D-LVA), which is statistically significant. In fact, the volume of the aneurysm in systole (or the surface area in systole) represents a characteristic determinant of dyskinetic aneurysms. With the loss of volume during systole, as expressed in D-LVA, there is a decrease in chamber ejection pressure proportionate to the size of the aneurysm, confirming the comparison with the mentioned biomechanical model (Holger Hadland) [7]. Indeed, the size of the volume of D-LVA in systole reflects the level of decrease in left ventricular ejection pressure. The volume of the aneurysm increases in systole in D-LVA compared to the diastolic value, while it decreases in akinetic ones. Holger Hadland and colleagues [7] demonstrated the effect of A-LVA and D-LVA on left ventricular function. It is clear that in A-LVA, the size and volume of the aneurysm do not affect the chamber and aortic curve of ejection pressure, and such aneurysms do not cause excessive mechanical defects in chamber function. Conversely, in the presence of a D-LVA, the ejection pressure of the left ventricle is reduced proportionally to the size of the D-LVA [7]. This results in volume loading of the chamber, or volume stress, and a reduction in chamber functional capacity. Sobkowitz and colleagues [21] established echocardiographic criteria based on the ratio of the aneurysm area to the end-diastolic area of the left ventricle and the ratio of the aneurysm volume to the LVEDV. Higher values above 0.4 indicate a large aneurysm, while lower values indicate a small aneurysm. The mean value of the proportional ratio LVA-area/LVED-area was 0.38, and the ratio LVA-volume/LVEDV was 0.35. Matsumoto and colleagues [2], from 23 analyzed patients with aneurysms, established echocardiographic criteria that a ratio of the aneurysm area to the left ventricle area determined from four chambers of 0.3 or higher is indicative of a large aneurysm.

Comparing the parameters of aneurysm size between A-LVA and D-LVA, our study concludes that the ratio of aneurysm volume to LVEDV is of approximate values (0.24 in A-LVA and 0.27 in D-LVA). This ratio is higher in systole in D-LVA (0.38 vs. 0.28 in A-LVA) due to the statistically larger volume of the aneurysm in systole (LVAVs) in D-LVA. Similarly, the ratio of aneurysm surface area to LVEDA is of approximate value (0.38 in A-LVA and 0.35 in D-LVA). Their ratio is higher in systole with the presence of a D-LVA (0.43) compared to 0.37 in A-LVA. Comparing the volume and surface area of the left ventricular aneurysm in systole and diastole, there are statistically significantly larger LVAVs and LVESAs with the presence of a D-LVA compared to an A-LVA. The LVAVs and LVAAs are higher due to the paradoxical movement of the wall of the D-LVA. Therefore, parameters for assessing aneurysm size (ratio of aneurysm volume to left ventricular volume and ratio of aneurysm surface area to left ventricular surface area) are imprecise if not determined in systole, especially for D-LVA. The ratio of the surface area and volume of the aneurysm to the surface area and volume of the left ventricle in diastole shows no difference between D-LVA and A-LVA. Therefore, they can be considered valid parameters for assessing aneurysm size, regardless of whether it is a D-LVA or an A-LVA.

The question arises about establishing more precise criteria for defining a large aneurysm anatomically. Two numerical ratios of aneurysm volume and surface area to left ventricular volume and surface area have been proposed in the literature: 0.4 [21] and a surface area ratio of 0.3 [2]. According to the results of our study, a large aneurysm is defined by a volume–surface ratio of the aneurysm to the left ventricle volume–surface of 0.3 or higher, independently of the phase of the cardiac cycle, which is consistent with the criteria set by Matsumoto and colleagues [2]. These conclusions are supported by experimental studies. An aneurysm that affects 32% of the chamber surface led to mechanical disturbances, especially functional ones, in reducing left ventricular ejection pressure in dyskinetic aneurysms [7]. An artificially induced aneurysm in experimental animals, reducing 30% of the left ventricular end-diastolic volume (LVAV/LVEDV ratio of 0.30), resulted in an ejection fraction (EF) of about 35% [22]. According to mechanical theory, the LVAV reflects the extent of myocardial injury. In this sense, absent (“conflicting”) movements of the left ventricular aneurysm region reduce the global contractility of the left ventricle. On the other hand, LVAV affects the volume load of the left ventricle, so LVAV can be used as a parameter to assess heart function. Therefore, a LVAV/LVEDV ratio reflects cardiac output to some extent. A larger LVAV/LVEDV ratio reflects a lower cardiac output, as the aneurysm cannot empty during systole and can only expand slightly. Thus, left ventricular ejection is less efficient, leading to a decrease in left ventricular EF.

It is known that the development of left ventricular aneurysm correlates with worsening heart function [23,24], as the left ventricular aneurysm reduces the percentage of functional myocardium contributing to left ventricular ejection. The paradoxical expansion of the aneurysm wall during systole (dyskinesia) increases volumetric load or volume stress on the contractile segments of the left ventricle, leading to greater impairment of left ventricular function in comparison to patients with akinetic aneurysms [25]. The results of our study show that the average EF in the A-LVA group was 28.18%, significantly lower at 21.25% in the D-LVA group. LVEDV was increased in both groups but more so in the D-LVA group, which is indirect evidence that there is more pronounced volume stress on the non-aneurysmal contractile part of the left ventricle in D-LVA, hence the lower EF value. High LVEDV has also been observed in other studies among D-LVA [25]. Stroke volume was approximately reduced in both groups (54.03 mL in A-LVA compared to 57.45 mL in D-LVA), reflecting the reduced percentage of functional myocardium contributing to left ventricular ejection.

Patients with aneurysms represent a heterogeneous group, as reports include mixed morphological types ranging from true aneurysms and small post-infarction aneurysms to true and globally dilated cardiomyopathies [17]. Therefore, it is not always possible to compare the data from individual studies. In most scientific studies, after surgical treatment of an aneurysm, there is an average increase in EF of 10–14%. The results of the EF increase are similar when it comes to D-LVA, whereas this trend is not manifested in A-LVA. LVEDV significantly decreases postoperatively in the D-LVA group compared to the A-LVA group [25]. LVEDV is an important determinant of surgical outcome in patients with LVA, as the goal of aneurysmectomy is to reduce LVEDV and thereby regional wall tension [26].

## 5. Conclusions

Echocardiographic diagnostics as the primary imaging technique clearly defines A-LVA and D-LVA, aneurysm size, hemodynamic impact in terms of the volume–surface area ratio of the aneurysm to the left ventricle, stroke volume, and ejection fraction. The echocardiographically determined volume size of D-LVA corresponds to the level of decrease in ventricular ejection pressure. The degree of reduction in ejection fraction reflects the magnitude of volumetric stress on non-aneurysmal segments of the left ventricle, particularly evident in D-LVA. The stroke volume size reflects the extent of reduction in the contractile (aneurysmal) portion of the left ventricle in both types of aneurysms. The prognostic value of individual parameters in defining left ventricular aneurysms is of great importance in determining treatment modalities, selecting surgical techniques, and postoperative status.

## Figures and Tables

**Table 1:** Values of aneurysm volume in diastole (LVAVd) and systole (LVAVs), aneurysm surface area in diastole (LVAAd) and systole (LVAAs), end-diastolic (LVEDV) and end-systolic left ventricular volume (LVESV), and end-diastolic (LVEDA) and end-systolic left ventricular surface area (LVESA) in akinetic and dyskinetic aneurysms.

	A-LVA (*n* = 34)	D-LVA (*n* = 20)	*p*-Value
LVAVd (mL)	68.79 ± 54.37	83.05 ± 65.09	0.393
LVAVs (mL)	51.54 ± 53.09	94.07 ± 74.66	0.009
LVAAd (cm^2^)	19.74 ± 9.68	21.67 ± 11.42	0.512
LVAAs (cm^2^)	16.41 ± 8.58	23.22 ± 11.73	0.018
LVEDV (mL)	261.71 ± 109.93	286.40 ± 102.27	0.417
LVESV (mL)	207.68 ± 201.34	228.95 ± 100.44	0.662
LVEDA (cm^2^)	53.50 ± 16.16	57.78 ± 11.74	0.307
LVESA (cm^2^)	42.76 ± 14.11	50.79 ± 13.33	0.045

LVAVd—left ventricular aneurysm volume in diastole; LVAVs—left ventricular aneurysm volume in systole; LVAAd—left ventricular aneurysm surface area in diastole; LVAAs—left ventricular aneurysm surface area in systole; LVEDV—left ventricular end diastolic volume; LVESV—left ventricular end systolic volume; LVEDA—left ventricular end diastolic surface area; LVESA—left ventricular end systolic surface area.

**Table 2:** Results of the ratio of aneurysm volume to left ventricular volume in diastole (LVAVd/LVEDV) and systole (LVAVs/LVESV) and the ratio of aneurysm surface area to left ventricular surface area in diastole (LVAAd/LVEDA) and systole (LVAAs/LVESA) for akinetic (A-LVA) and dyskinetic (D-LVA) aneurysms.

	A-LVA (*n* = 34)	D-LVA (*n* = 20)	*p*-Value
LVAVd/LVEDV	0.24 ± 0.11	0.27 ± 0.17	0.454
LVAVs/LVESV	0.28 ± 0.15	0.38 ± 0.23	0.052
LVAAd/LVEDA	0.38 ± 0.16	0.35 ± 0.14	0.570
LVAAs/LVESA	0.37 ± 0.12	0.43 ± 0.16	0.122

LVAVd/LVEDV—ratio of left ventricular volume in diastole and left ventricular end diastolic volume; LVAVs/LVESV—ratio of left ventricular volume in systole and left ventricular end systolic volume; LVAAd/LVEDA—left ventricular aneurysm surface area in diastole and left ventricular end diastolic surface area; LVAAs/LVESA—left ventricular aneurysm surface area in systole and left ventricular end systolic surface area.

**Table 3:** Values of ejection fraction (EF), end-diastolic (LVEDV), and end-systolic volume (LVESV) of the left ventricle, and stroke volume (SV) in akinetic (A-LVA) and dyskinetic (D-LVA) aneurysms.

	A-LVA (*n* = 34)	D-LVA (*n* = 20)	*p*-Value
EF (%)	28.18 ± 11.91	21.25 ± 11.92	0.044
LVEDV (mL)	261.71 ± 109.93	286.40 ± 102.27	0.417
LVESV (mL)	207.68 ± 201.34	228.95 ± 100.44	0.662

EF—ejection fraction; LVEDV—left ventricular end diastolic volume; LVESV—left ventricular end systolic volume.

## Data Availability

Data are available upon request.

## Abbreviations

A	LVA akinetic left ventricular aneurysm
D	LVA dyskinetic left ventricular aneurysm
EF	ejection fraction
SV	stroke volume
LV	left ventricular
LVA	left ventricular aneurysm
LVAA	left ventricular aneurysm surface area
LVAAs	left ventricular aneurysm surface area in systole
LVAAd	left ventricular aneurysm surface area in diastole
LVAV	left ventricular aneurysm volume
LVAVs	left ventricular aneurysm volume in systole
LVAVd	left ventricular aneurysm volume in diastole
LVAAS	left ventricular aneurysm surface area in systole
LVESV	left ventricular end systolic volume
LVEDV	left ventricular end diastolic volume
LVEDA	left ventricular end diastolic surface area
LVESA	left ventricular end systolic surface area
